# Ancient coexistence of norepinephrine, tyramine, and octopamine signaling in bilaterians

**DOI:** 10.1186/s12915-016-0341-7

**Published:** 2017-01-30

**Authors:** Philipp Bauknecht, Gáspár Jékely

**Affiliations:** Max Planck Institute for Developmental Biology, Spemannstrasse 35, 72076 Tübingen, Germany

**Keywords:** Octopamine, Tyramine, Norepinephrine, Noradrenaline, GPCR evolution, Neurotransmitter, *Saccoglossus*, *Platynereis*, *Priapulus*, Xenacoelomorpha

## Abstract

**Background:**

Norepinephrine/noradrenaline is a neurotransmitter implicated in arousal and other aspects of vertebrate behavior and physiology. In invertebrates, adrenergic signaling is considered absent and analogous functions are performed by the biogenic amines octopamine and its precursor tyramine. These chemically similar transmitters signal by related families of G-protein-coupled receptors in vertebrates and invertebrates, suggesting that octopamine/tyramine are the invertebrate equivalents of vertebrate norepinephrine. However, the evolutionary relationships and origin of these transmitter systems remain unclear.

**Results:**

Using phylogenetic analysis and receptor pharmacology, here we have established that norepinephrine, octopamine, and tyramine receptors coexist in some marine invertebrates. In the protostomes *Platynereis dumerilii* (an annelid) and *Priapulus caudatus* (a priapulid), we have identified and pharmacologically characterized adrenergic α1 and α2 receptors that coexist with octopamine α, octopamine β, tyramine type 1, and tyramine type 2 receptors. These receptors represent the first examples of adrenergic receptors in protostomes. In the deuterostome *Saccoglossus kowalevskii* (a hemichordate), we have identified and characterized octopamine α, octopamine β, tyramine type 1, and tyramine type 2 receptors, representing the first examples of these receptors in deuterostomes. *S. kowalevskii* also has adrenergic α1 and α2 receptors, indicating that all three signaling systems coexist in this animal. In phylogenetic analysis, we have also identified adrenergic and tyramine receptor orthologs in xenacoelomorphs.

**Conclusions:**

Our results clarify the history of monoamine signaling in bilaterians. Given that all six receptor families (two each for octopamine, tyramine, and norepinephrine) can be found in representatives of the two major clades of Bilateria, the protostomes and the deuterostomes, all six receptors must have coexisted in the last common ancestor of the protostomes and deuterostomes. Adrenergic receptors were lost from most insects and nematodes, and tyramine and octopamine receptors were lost from most deuterostomes. This complex scenario of differential losses cautions that octopamine signaling in protostomes is not a good model for adrenergic signaling in deuterostomes, and that studies of marine animals where all three transmitter systems coexist will be needed for a better understanding of the origin and ancestral functions of these transmitters.

**Electronic supplementary material:**

The online version of this article (doi:10.1186/s12915-016-0341-7) contains supplementary material, which is available to authorized users.

## Background

**Fig. 1:**
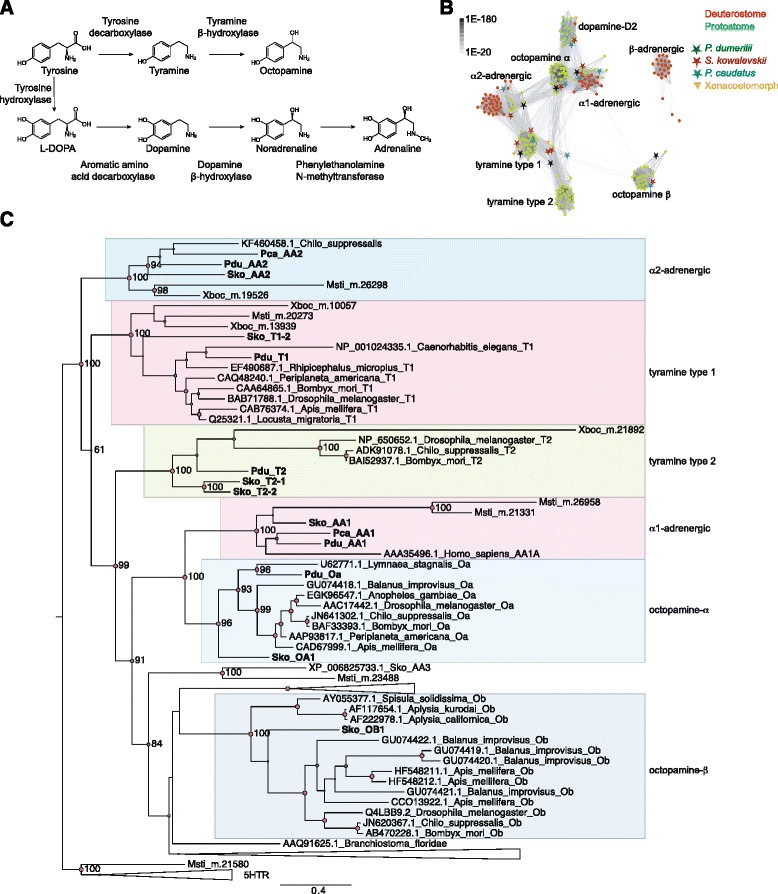
Biosynthesis of monoamines and phylogeny of adrenergic, tyramine, and octopamine G-protein-coupled receptor (GPCR) sequences. **a** Biosynthesis of tyramine, octopamine, norepinephrine, and epinephrine from tyrosine. The enzymes catalyzing the reaction steps are indicated. **b** Sequence-similarity-based cluster map of bilaterian octopamine, tyramine, and adrenergic GPCRs. Nodes correspond to individual GPCRs and are colored based on taxonomy. Edges correspond to BLAST connections of *P* value >1e−70. **c** Simplified phylogenetic tree of bilaterian adrenergic, tyramine, and octopamine GPCR sequences. The tree is rooted on 5HT receptors (5HTR). *Abbreviations*: Pdu *P. dumerilii*, Pca *P. caudatus*, Sko *S. kowalevskii*, Msti *M. stichopi*, Xboc *X. bocki*

Norepinephrine is a major neurotransmitter in vertebrates with a variety of functions, including roles in promoting wakefulness and arousal [1], regulating aggression [2], and autonomic functions such a heart beat [3]. Signaling by the monoamine octopamine in protostome invertebrates is often considered equivalent to vertebrate adrenergic signaling [4], with analogous roles in promoting aggression and wakefulness in flies [5, 6], and the regulation of heart rate in annelids and arthropods [7, 8]. Octopamine is synthesized from tyramine (Fig. 1a) which itself also acts as a neurotransmitter or neuromodulator in arthropods and nematodes [4, 9–15]. Octopamine and norepinephrine are chemically similar, are synthesized by homologous enzymes [16, 17], and signal by similar but not orthologous G-protein-coupled receptors (GPCRs) [4, 18].

Tyramine also signals via non-orthologous receptors in invertebrates and vertebrates. In insects and nematodes, tyramine signals via a GPCR that is related to octopamine receptors [12, 19]. In vertebrates, tyramine is only present at low levels and signals via the trace-amine receptors, a vertebrate-specific GPCR family only distantly related to the invertebrate tyramine receptors [20, 21]. Given these differences, the precise evolutionary relationships of these monoamine signaling systems are unclear.

The evolution of neurotransmitter systems has been analyzed by studying the distribution of monoamines or biosynthetic enzymes in different organisms [22]. This approach has limitations, however, because some of the biosynthetic enzymes are not specific to one substrate [16] and because trace amounts of several monoamines are found across many organisms, even if specific receptors are often absent [22]. For example, even if invertebrates can synthesize trace amounts of norepinephrine, these are not considered to be active neuronal signaling molecules, because the respective receptors are lacking. Consequently, the presence of specific monoamine receptors is the best indicator that a particular monoamine is used in neuronal signaling [11, 23].

To clarify the evolutionary history of adrenergic, octopamine, and tyramine signaling in animals, we undertook a comparative phylogenetic and pharmacological study of these receptor families in bilaterians. Bilaterians—animals with bilateral symmetry—comprise protostomes, deuterostomes, and xenacoelomorphs [24]. Deuterostomes include chordates and ambulacrarians (hemichordates and echinoderms), and protostomes are formed by the clades Ecdysozoa, Lophotrochozoa (Spiralia), and Chaetognatha. Ecdysozoa includes arthropods, nematodes, priapulids and other phyla. Lophotrochozoa includes annelids, mollusks, and other, mostly marine groups. Xenacoelomorpha, a group including acoel flatworms, nemertodermatids, and *Xenoturbella*, has been proposed to belong to the deuterostomes, or represent a sister group to all remaining bilaterians [25–27]. Here, we have attempted to establish the orthologous relationships of adrenergic, octopamine, and tyramine receptors across bilaterians. We found that six receptor families originated at the base of the bilaterian tree. We then pharmacologically characterized adrenergic receptors from an annelid and a priapulid, and octopamine and tyramine receptors from an annelid and a hemichordate. The broad phylogenetic sampling and comparative pharmacology paint a richer picture of the evolution of these receptors, characterized by ancestral coexistence and multiple independent losses.

## Results

Using database searches, sequence-similarity-based clustering, and phylogenetic analysis, we reconstructed the phylogeny of α1, α2, and β adrenergic, octopamine α, octopamine β, and tyramine type-1 and type-2 receptors. Each family formed well-resolved clusters in a sequence-similarity-based clustering analysis and well-supported clades in molecular phylogenetic analysis (Fig. 1b, c and Additional file 1).

We identified several invertebrate GPCR sequences that were similar to vertebrate adrenergic α1 and α2 receptors (Fig. 1b, c). An adrenergic α1 receptor ortholog is present in the sea urchin *Strongylocentrotus purpuratus*. Adrenergic α1 and α2 receptors were both present in *Saccoglossus kowalevskii,* a hemichordate deuterostome (Fig. 1b, c and Additional files 1, 2, and 3), as previously reported [28]. We also identified adrenergic α1 and α2 receptor orthologs in annelids and mollusks (members of the Lophotrochozoa), including *Aplysia californica,* and in the priapulid worm *Priapulus caudatus* (member of the Ecdysozoa) (Fig. 1b, c and Additional files 1, 2, and 3). Adrenergic α receptors are also present in a few arthropods, including the crustacean *Daphnia pulex* and the moth *Chilo suppressalis* (the *Chilo* α2 receptor was first described as an octopamine receptor [29]), but are absent from most other insects (Additional files 1, 2, and 3). Adrenergic α2 receptors are also present in the xenacoelomorphs *Xenoturbella bocki* and *Meara stichopi. M. stichopi* also has two adrenergic α1 receptor orthologs (Fig. 1c and Additional files 1, 2, and 3).

The identification of adrenergic α1 and of α2 receptor orthologs in ambulacrarians, lophotrochozoans, ecdysozoans, and xenacoelomorphs indicates that both families were present in the bilaterian last common ancestor.

Adrenergic β receptors are found in chordates, including urochordates and cephalochordates. In addition, we identified an adrenergic β receptor ortholog in the xenacoelomorph *M. stichopi* (Additional file 4). If xenacoelomorphs are sister to all remaining bilaterians, then this receptor family also originated at the base of Bilateria and was lost from all protostomes.

**Fig. 2:**
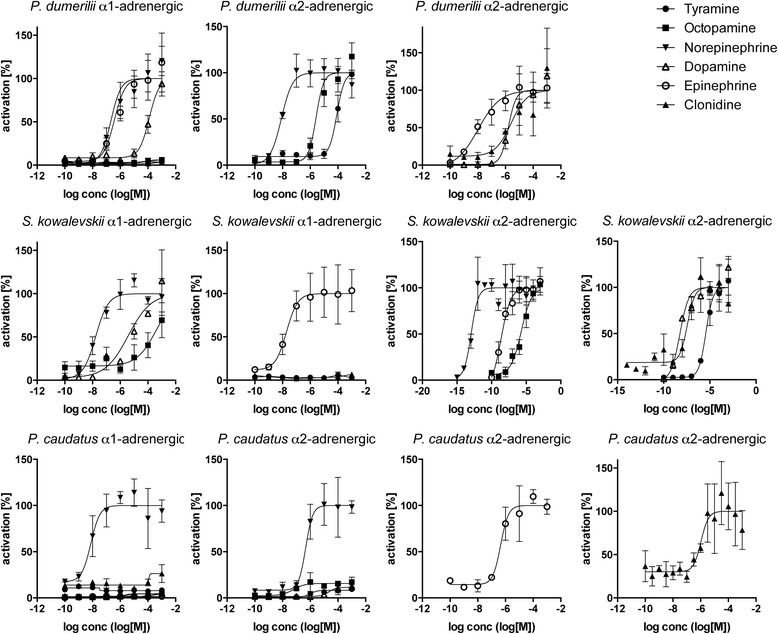
Dose–response curves of adrenergic G-protein-coupled receptors from *Platynereis dumerilii*, *Priapulus caudatus*, and *Saccoglossus kowalevskii* treated with varying concentrations of ligand. Data, representing luminescence units relative to the maximum of the fitted dose–response curves, are shown as mean ± standard error of the mean (n = 3). Half maximal effective concentration (EC_50_) values and significance values are listed in Table 1

To characterize the ligand specificities of these putative invertebrate adrenergic receptors, we cloned them from *S. kowalevskii*, *Priapulus caudatus*, and the marine annelid *Platynereis dumerilii*. We performed in vitro GPCR activation experiments using a Ca^2+^-mobilization assay [30, 31]. We found that norepinephrine and epinephrine activated both the adrenergic α1 and α2 receptors from all three species with half maximal effective concentration (EC_50_) values in the high nanomolar range or lower. In contrast, tyramine, octopamine, and dopamine were either inactive or only activated the receptors at concentrations approximately two orders of magnitude higher (Fig. 2, Table 1). These phylogenetic and pharmacological results collectively establish these invertebrate receptors as bona fide adrenergic α receptors.

**Table 1:** Half maximal effective concentration (EC_50_) (M) and half maximal inhibitory concentration (IC_50_) (M) values of all tested G-protein-coupled receptors with the indicated ligands or inhibitors

EC50 (M)/IC50 (M)	Tyramine	Octopamine	Clonidine	Norepinephrine	Dopamine	Epinephrine	Yohimbine	Mianserin
*Platynereis dumerilii* α1-adrenergic	inactive	inactive	inactive	2.1E − 07***	1.2E − 04***	3.7E − 07 ns	4.4E − 06	3.7E − 06
95% CI				1.0E − 007 to 4.2E − 007	2.7E − 005 to 0.00056	1.3E − 007 to 1.1E − 006	2.3E − 006 to 8.2E − 006	1.9E − 006 to 7.2E − 006
*P. dumerilii* α2-adrenergic	8.4E − 05	2.7E − 06***	2.6E − 06	8.2E − 09***	1.6E − 06	1.1E − 08 ns	5.7E − 06	2.5E − 05
95% CI	2.8E − 005 to 0.00024	6.683E − 007 to 1.0E − 005	2.4E − 007 to 2.7E − 005	5.7E − 009 to 1.1E − 008	8.3E − 007 to 3.2E − 006	5.0E − 009 to 2.2E − 008	3.5E − 006 to 9.1E − 006	1.2E − 005 to 5.1E − 005
*S. kowalevskii* α1-adrenergic	inactive	inactive	inactive	1.7E − 08***	3.8E − 06***	1.9E − 08 ns	1.3E − 05	4.5E − 06
95% CI				1.0E − 008 to 2.7E − 008	1.9E − 007 to 7.4E − 005	9.0E − 009 to 4.1E − 008	7.6E − 006 to 2.2E − 005	1.6E − 006 to 1.1E − 005
*S. kowalevskii* α2-adrenergic	3.7E − 06	1.9E − 06	3.6E − 08	1.2E − 13***	5.6E − 09	2.3E − 09***	3.3E − 07	inactive
95% CI	2.0E − 006 to 6.8E − 006	2.5E − 007 to 1.4E − 005	6.7E − 009 to 1.9E − 007	6.7E − 014 to 1.9E − 013	3.3E − 009 to 9.4E − 009	1.1E − 009 to 4.6E − 009	2.6E − 007 to 4.0E − 007	
*Priapulus caudatus* α1-adrenergic	inactive	inactive	inactive	**7.5E − 09**	inactive	inactive	inactive	inactive
95% CI				4.0E − 009 to 1.3E − 008				
*P. caudatus* α2-adrenergic	inactive	inactive	1.1E − 06 * p = 0.021	4.7E − 07*	inactive	**4.5E − 07** ns	inactive	9.8E − 07
95% CI			4.5E − 007 to 2.4E − 006	1.7E − 007 to 1.2E − 006		1.8E − 007 to 1.0E − 006		4.3E − 007 to 2.2E − 006
*P. dumerilii* Tyramine-1	1.1E − 08***	2.7E − 06***	2.1E − 06	1.7E − 05	7.8E − 06	3.1E − 05	2.1E − 06	4.7E − 05
95% CI	7.6E − 009 to 1.6E − 008	1.1E − 006 to 6.1E − 006	1.0E − 006 to 4.1E − 006	1.0E − 005 to 2.8E − 005	1.5E − 006 to 3.9E − 005	9.8E − 006 to 9.9E − 005	7.0E − 007 to 6.0E − 006	1.7E − 005 to 0.00012
*P. dumerilii* Tyramine-2	7.0E − 09***	7.8E − 07***	5.3E − 06	1.1E − 04	3.9E − 06	4.8E − 05	5.4E − 05	6.4E − 06
95% CI	3.0E − 009 to 1.6E − 008	3.8E − 007 to 1.5E − 006	2.1E − 006 to 1.3E − 005	2.9E − 005 to 0.00038	2.1E − 006 to 7.0E − 006	8.6E − 006 to 0.00026	3.6E − 005 to 7.9E − 005	3.9E − 006 to 1.0E − 005
*S. kowalevskii* Tyramine-1	**8.6E − 05** ns	inactive	2.9E − 04 n.s.	inactive	0.57	inactive	1.7E − 06	1.7E − 05
95% CI	2.8E − 005 to 0.00025		0.00013 to 0.00065		very wide	2.1E − 006 to 0.00017	7.1E − 007 to 3.9E − 006	7.7E − 006 to 3.7E − 005
*S. kowalevskii* Tyramine-2A	1.0E − 09***	8.6E − 08***	1.4E − 06	inactive	7.2E − 08	inactive	inactive	1.6E − 04
95% CI	6.6E − 010 to 1.5E − 009	4.0E − 008 to 1.8E − 007	7.4E − 007 to 2.6E − 006		1.4E − 008 to 3.5E − 007			5.4E − 008 to 0.47
*S. kowalevskii* Tyramine-2B	5.9E − 09***	1.6E − 06***	1.6E − 05	1.2E − 04	1.4E − 06	2.8E − 05	2.1E − 05	1.9E − 05
95% CI	2.4E − 009 to 1.4E − 008	6.5E − 007 to 3.7E − 006	6.2E − 006 to 4.0E − 005	3.6E − 005 to 0.00036	9.0E − 007 to 2.2E − 006	5.1E − 006 to 0.00015	1.1E − 005 to 3.6E − 005	1.1E − 005 to 3.0E − 005
*P. dumerilii* Octopamine α	1.3E − 05	2.6E − 07*	1.4E − 07 n.s.	3.5E − 06* *P* = 0.003	inactive	8.8E − 06	9.0E − 09	1.6E − 06
95% CI	4.2E − 006 to 4.1E − 005	8.4E − 008 to 7.7E − 007	6.7E − 008 to 3.0E − 007	1.8E − 006 to 6.7E − 006		2.5E − 006 to 3.0E − 005	4.1E − 009 to 1.9E − 008	9.7E − 007 to 2.6E − 006
*S. kowalevskii* Octopamine α	1.7E − 05	6.9E − 07*	1.6E − 07 * p = 0.048	5.3E − 05	2.6E − 04	1.8E − 05	7.8E − 06	2.2E − 05
95% CI	3.0E − 006 to 9.5E − 005	1.8E − 007 to 2.4E − 006	7.6E − 008 to 3.5E − 007	1.5E − 005 to 0.00018	3.4E − 006 to 0.02	7.1E − 006 to 4.7E − 005	3.1E − 006 to 1.8E − 005	1.2E − 005 to 3.6E − 005
*S. kowalevskii* Octopamine β	inactive	6.4E − 08***	inactive	3.5E − 06***	inactive	inactive	1.6E − 04	6.4E − 06
95% CI		4.0E − 008 to 1.0E − 007		1.4E − 006 to 8.1E − 006			1.0E − 005 to 0.0023	3.1E − 006 to 1.3E − 005

The most effective natural ligand for each receptor is shown in bold. 95% confidence intervals (*CI*) for the EC_50_ (M)/IC_50_ (M) values are given in every second line. The lowest EC_50_ value for each receptor was compared to the next lowest one using the extra sum-of-squares F test. *P < 0.05; ***P < 0.0001; *ns* not significant. Significance values are shown for the compared pairs

**Fig. 3:**
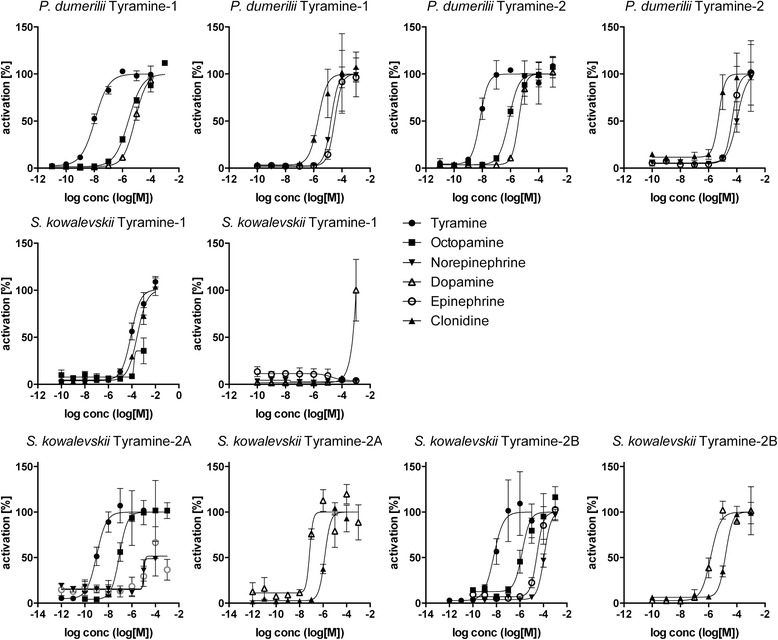
Dose–response curves of tyramine G-protein-coupled receptors from *Platynereis dumerilii* and *Saccoglossus kowalevskii* treated with varying concentrations of ligand. Data, representing luminescence units relative to the maximum of the fitted dose–response curves, are shown as mean ± standard error of the mean (n = 3). EC_50_ values and significance values are listed in Table 1

**Fig. 4:**
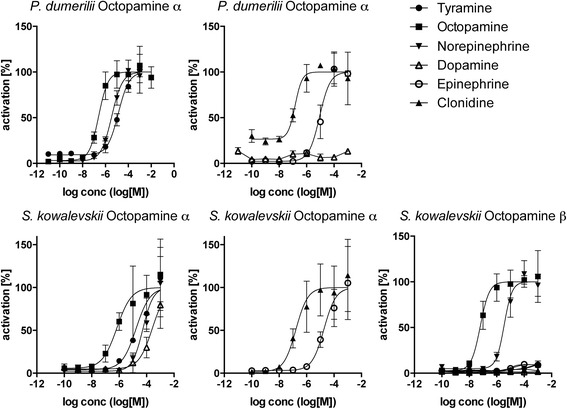
Dose–response curves of octopamine G-protein-coupled receptors from *Platynereis dumerilii* and *Saccoglossus kowalevskii* treated with varying concentrations of ligand. Data, representing luminescence units relative to the maximum of the fitted dose–response curves, are shown as mean ± standard error of the mean (n = 3). Half maximal effective concentration (EC_50_) values and significance values are listed in Table 1

To investigate if adrenergic signaling coexists with octopamine and tyramine signaling in protostomes, we searched for octopamine and tyramine receptors in *Platynereis dumerilii* and *Priapulus caudatus*. In phylogenetic and clustering analyses, we identified orthologs for tyramine type 1 and type 2 and octopamine α and β receptors in both species (Fig. 1b, c and Additional files 5, 6, 7, and 8). We performed activation assays with the *Platynereis dumerilii* receptors. The tyramine type 1 and type 2 receptors orthologs were preferentially activated by tyramine with EC_50_ values in the nanomolar range (Fig. 3, Table 1). The *Platynereis dumerilii* octopamine α receptor was activated by octopamine at a lower concentration than by tyramine and dopamine (Fig. 4, Table 1). The *Platynereis dumerilii* octopamine β receptor was not active in our assay. These results show that specific receptor systems for norepinephrine, octopamine, and tyramine coexist in *Platynereis dumerilii* and very likely also *Priapulus caudatus*.

When did tyramine and octopamine signaling originate? To answer this, we surveyed available genome sequences for tyramine and octopamine receptors. As expected, we identified several receptors across the protostomes, including ecdysozoans and lophotrochozoans (Additional files 5, 6, 7, and 8). We also identified receptors for tyramine, but not octopamine, in xenacoelomorphs. However, chordate genomes lacked orthologs of these receptors. Strikingly, we identified tyramine type 1 and 2 and octopamine α and β receptor orthologs in the genome of the hemichordate *S. kowalevskii* (Fig. 1b, c, Additional files 5, 6, 7, and 8). In phylogenetic analyses, we recovered at least one *S. kowalevskii* sequence in each of the four receptor clades (one octopamine α, one octopamine β, two tyramine type 1, and two tyramine type 2 receptors), establishing these sequences as deuterostome orthologs of these predominantly protostome GPCR families (Additional files 5, 6, 7, and 8).

We cloned the candidate *S. kowalevskii* tyramine and octopamine receptors and performed ligand activation experiments. The *S. kowalevskii* type 2 receptors were preferentially activated by tyramine in the nanomolar range. The type 1 receptor was only activated at higher ligand concentrations. The octopamine α and β receptors were preferentially activated by octopamine in the nanomolar range (Figs 3 and 4, Table 1). These data show that octopamine and tyramine signaling also coexist with adrenergic signaling in this deuterostome, as in *Platynereis dumerilii* and *Priapulus caudatus*. The presence of tyramine signaling in *S. kowalevskii* is also supported by the phylogenetic distribution of tyrosine decarboxylase, a specific enzyme for tyramine synthesis [32]. Tyrosine decarboxylase is present in protostomes and *S. kowalevskii* but is absent from other deuterostomes (Additional file 9). In mammals, aromatic amino acid decarboxylases are involved in synthesizing low amounts of tyramine [33].

We also tested the α adrenergic agonist clonidine and the GPCR antagonists mianserin and yohimbine on several receptors from all three species. These chemicals did not show specificity for any of the receptor types, suggesting these chemicals may not be useful for studying individual biogenic amine receptors in vivo (Table 1 and Additional file 10).

## Discussion

**Fig. 5:**
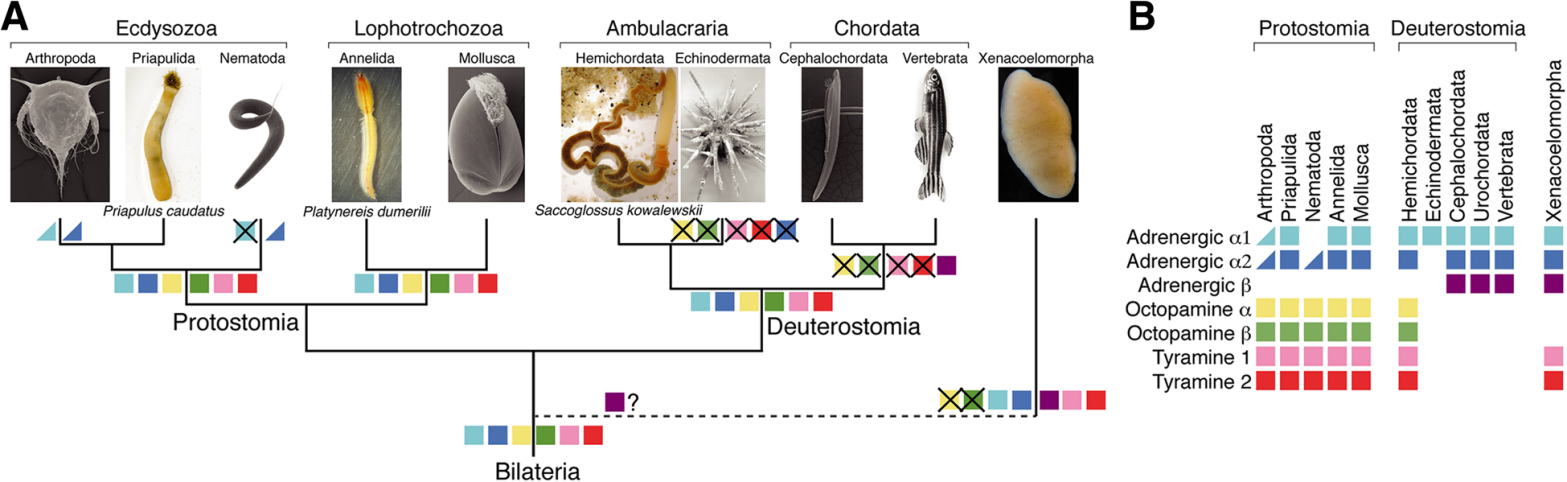
Evolution of adrenergic, octopamine, and tyramine signaling in bilaterians. **a** Phylogenetic tree of major clades of bilaterian animals with the presence/loss of specific G-protein-coupled receptor (GPCR) families indicated. **b** Phyletic distribution of adrenergic, octopamine, and tyramine GPCR families across major bilaterian clades. Half squares mean losses in a large number of species in a phylum

The discovery of adrenergic signaling in some protostomes and xenacoelomorphs and octopamine and tyramine signaling in a deuterostome changes our view on the evolution of monoamine signaling in bilaterians (Fig. 5). It is clear from the phylogenetic distribution of orthologous receptor systems that at least six families of octopamine, tyramine, and adrenergic receptors were present in the bilaterian last common ancestor (Additional file 11). These include the adrenergic α1 and α2 receptors, the tyramine type 1 and type 2 receptors, and the octopamine α and β receptors. From the six ancestral families, the octopamine and tyramine receptors have been lost from most deuterostomes, and the adrenergic receptors from most ecdysozoans. Interestingly, the xenacoelomorph *M. stichopi* also has an adrenergic β receptor, representing the only ortholog outside chordates. Octopamine α receptors have likely been lost from xenacoelomorphs, given that the split of the six receptor families (four with well-resolved xenacoelomorph sequences) pre-dated the divergence of the main lineages of bilaterians (Fig. 1c).

Although we performed the receptor activation assays in a heterologous system that might not mimic the in vivo situation very well, we found clear evidence of ligand preferences for each receptor. In general, there was two orders of magnitude difference in the EC_50_ values between the best ligand and other related ligands for the same receptor measured under the same conditions. We consider these in vitro ligand preferences as indicative of the physiological ligands for these receptors. Furthermore, there was high congruence between the in vitro ligand specificities and the phylogenetic placement of the different classes of receptors, further strengthening our receptor-type assignments. The most potent ligand of all six orthologous receptor families we analyzed was the same across protostomes and deuterostomes, indicating the evolutionary stability of ligand–receptor pairs, similar to the long-term stability of neuropeptide GPCR ligand–receptor pairs [34, 35].

Understanding the ancestral role of these signaling systems and why they may have been lost differentially in different animal groups will require functional studies in organisms where all three neurotransmitter systems coexist.

## Conclusions

We have established the coexistence of adrenergic, octopaminergic, and tyraminergic signaling in the deuterostome *S. kowalevskii* and the protostomes *Platynereis dumerilii* and *Priapulus caudatus.* Signaling by norepinephrine in vertebrates has often been considered as equivalent to signaling by octopamine in invertebrates. Our results change this view and show that these signaling systems coexisted ancestrally and still coexist in some bilaterians. The extent of functional redundancy in species where all six receptor systems coexist will require experimental studies. It may be that some of these monoamines ancestrally had partially overlapping roles. In that case, following the loss of a receptor, functions associated with that ligand–receptor pair may have been taken over by another pair. However, regardless of such potential shifts in function, it is clear that octopamine signaling in invertebrates and adrenergic signaling in vertebrates is not equivalent or homologous from an evolutionary point of view. This has important implications for our interpretation of comparative studies of the function of these neurotransmitter systems and their neural circuits. Our study also contributes to the understanding of nervous system evolution in bilaterians by revealing extensive losses during the history of one of the major classes of neurotransmitter systems.

## Methods

### Gene identification and receptor cloning

*Platynereis* protein sequences were collected from a *Platynereis* mixed-stage transcriptome assembly [36]. GPCR sequences from other species were downloaded from NCBI. GPCRs were cloned into pcDNA3.1(+) (Thermo Fisher Scientific, Waltham, MA, USA) as described before [31]. Forward primers consisted of a spacer (ACAATA) followed by a BamHI or EcoRI restriction site, the Kozak consensus sequence (CGCCACC), a start codon (ATG), and a sequence corresponding to the target sequence. Reverse primers consisted of a spacer (ACAATA), a NotI restriction site, a STOP codon, and a reverse complementary sequence to the target sequence. Primers were designed to end with a C or G with a 72 °C melting temperature. Polymerase chain reaction was performed using Phusion polymerase (New England Biolabs GmbH, Frankfurt, Germany). The sequences of all *Platynereis* GPCRs tested here were deposited in GenBank (accession numbers: α1-adrenergic receptor [GenBank: KX372342]; α2-adrenergic receptor [GenBank: KX372343], Tyramine-1 receptor [GenBank: KP293998]; Tyramine-2 receptor [GenBank: KU715093]; Octopamine α receptor [GenBank: KU530199]; Octopamine β receptor [GenBank: KU886229]). Tyramine receptor 1 has previously been published [31] as Pdu orphan GPCR 48. The GenBank accession numbers of the *S. kowalevskii* and *Priapulus caudatus* sequences tested are: *S. kowalevskii* α1-adrenergic [GenBank: ALR88680]; *S. kowalevskii* α2-adrenergic [GenBank: XP_002734932]; *Priapulus caudatus* α1-adrenergic [GenBank: XP_014662992]; *Priapulus caudatus* α2-adrenergic [GenBank: XP_014681069]; *S. kowalevskii* Tyramine-1 [GenBank: XP_002742354]; *S. kowalevskii* Tyramine-2A [GenBank: XP_002734062]; *S. kowalevskii* Tyramine-2B [GenBank: XP_006812999]; *S. kowalevskii* Octopamine α, [GenBank: XP_006823182]; and *S. kowalevskii* Octopamine β [GenBank: XP_002733926].

### Cell culture and receptor deorphanization

Cell culture assays were done as described before [31]. Briefly, CHO-K1 cells were kept in Ham’s F12 Nut Mix medium (Thermo Fisher Scientific) with 10% fetal bovine serum and penicillin-streptomycin (PenStrep, Thermo Fisher Scientific). Cells were seeded in 96-well plates (Thermo Fisher Scientific) at approximately 10,000 cells/well. After 1 day, cells were transfected with plasmids encoding a GPCR, the promiscuous Gα-16 protein [37], and a reporter construct GFP-apoaequorin [38] (60 ng each) using 0.375 μL of the transfection reagent TurboFect (Thermo Fisher Scientific). After 2 days of expression, the medium was removed and replaced with Hank’s Balanced Salt Solution (HBSS) supplemented with 1.8 mM Ca^2+^, 10 mM glucose, and 1 mM coelenterazine h (Promega, Madison, WI, USA). After incubation at 37 °C for 2 h, cells were tested by adding synthetic monoamines (Sigma, St. Louis, MO, USA) in HBSS supplemented with 1.8 mM Ca^2+^ and 10 mM glucose. Solutions containing norepinephrine, epinephrine, or dopamine were supplemented with 100 μM ascorbic acid to prevent oxidation. Luminescence was recorded for 45 s in a plate reader (BioTek Synergy Mx or Synergy H4; BioTek, Winooski, VT, USA). For inhibitor testing, the cells were incubated with yohimbine or mianserin (Sigma) for 1 h. Then, synthetic monoamines were added to yield in each case the smallest final concentration expected to elicit the maximal response in the absence of inhibitor, and luminescence was recorded for 45 s. Data were integrated over the 45-s measurement period. Data for dose–response curves were recorded as technical triplicates for each concentration. Measurements were performed from adjacent wells on the same plate to minimize variation introduced by cell seeding and transfection. Dose–response curves were fitted with a four-parameter curve using Prism 6 (GraphPad, La Jolla, CA, USA). The curves were normalized to the calculated upper plateau values (100% activation). The different EC_50_ values for each receptor were compared with the extra sum-of-squares F test in a pairwise manner using Prism 6.

### Bioinformatics

Protein sequences were downloaded from the NCBI. Redundant sequences were removed from the collection using CD-HIT [39] with an identity cutoff of 70%. Sequence cluster maps were created with CLANS2 [40] using the BLOSUM62 matrix and a *P*-value cutoff of 1e−70. For phylogenetic trees, protein sequences were aligned with MUSCLE [41]. Alignments were trimmed with TrimAI [42] in “Automated 1” mode. The best amino acid substitution model was selected using ProtTest 3 [43]. Maximum likelihood trees were calculated with RAxML [44] using the CIPRES Science Gateway [45] or with IQ-TREE and automatic model selection (http://www.iqtree.org/). Bootstrap analysis in RAxML was done and automatically stopped [46] when the Majority Rule Criterion (autoMRE) was met. The resulting trees were visualized with FigTree (http://tree.bio.ed.ac.uk/software/figtree/). The identifiers of deorphanized adrenergic, octopamine, and tyramine receptors [12, 29, 47–59] were tagged with _AA1, AA2, _Oa, _Ob, _T1, or _T2. The trees were rooted on 5HT receptors. The full phylogenetic tree is available in nexus format (Additional file 11).

## Additional files

Additional file 1: Maximum likelihood tree of adrenergic, octopamine, and tyramine receptors. Bootstrap support values are shown. This tree contains all investigated GPCRs. The tree was rooted on 5HT receptor sequences. Sub-trees are shown in Additional files 2, 3, 4, 5, 6, 7, and 8. (PDF 118 kb)
Additional file 2: Maximum likelihood tree of α1-adrenergic receptors. Bootstrap support values are shown for selected nodes. This tree is part of a larger tree containing all investigated GPCRs. (PDF 16992 kb)
Additional file 3: Maximum likelihood tree of α2-adrenergic receptors. Bootstrap support values are shown for selected nodes. This tree is part of a larger tree containing all investigated GPCRs. (PDF 17168 kb)
Additional file 4: Maximum likelihood tree of β-adrenergic receptors. Bootstrap support values are shown for some nodes of interest. This tree is part of a larger tree containing all investigated GPCRs. (PDF 759 kb)
Additional file 5: Maximum likelihood tree of tyramine type 1 receptors. Bootstrap support values are shown for selected nodes. This tree is part of a larger tree containing all investigated GPCRs. The identifiers of deorphanized tyramine receptors were tagged with _T1. (PDF 17028 kb)
Additional file 6: Maximum likelihood tree of tyramine type 2 receptors. Bootstrap support values are shown for selected nodes. This tree is part of a larger tree containing all investigated GPCRs. The identifiers of deorphanized tyramine receptors were tagged with _T2. (PDF 17007 kb)
Additional file 7: Maximum likelihood tree of octopamine-α receptors. Bootstrap support values are shown for selected nodes. This tree is part of a larger tree containing all investigated GPCRs. The identifiers of deorphanized octopamine receptors were tagged with _Oa. (PDF 16730 kb)
Additional file 8: Maximum likelihood tree of octopamine-β receptors. Bootstrap support values are shown for selected nodes. This tree is part of a larger tree containing all investigated GPCRs. The identifiers of deorphanized octopamine receptors were tagged with _Ob. (PDF 16730 kb)
Additional file 9: Maximum likelihood tree of tyrosine decarboxylase and aromatic amino acid decarboxylase enzymes. Bootstrap support values are shown for selected nodes. *P. dumerilii*, *P. caudatus*, and *S. kowalevskii* sequences are highlighted in color. The *Caenorhabditis elegans* tyrosine decarboxylase was experimentally shown to be required for tyramine biosynthesis [32]. (PDF 566 kb)
Additional file 10: Dose–response curves of adrenergic, tyramine, and octopamine receptors from *P. dumerilii*, *P. caudatus*, and *S. kowalevskii* treated with varying concentrations of inhibitors. Data, representing luminescence units relative to the maximum of the fitted dose–response curves, are shown as mean ± SEM (n = 3). IC_50_ values are listed in Table 1. (TIF 956 kb)
Additional file 11: Maximum likelihood tree of octopamine, tyramine, and adrenergic α receptors, in nexus format. (NEXUS 37 kb)
