# Supplementary figures and images for: Ancient coexistence of norepinephrine, tyramine, and octopamine signaling in bilaterians
Source: BMC Biol. 2017 Jan 30;15:6. doi: 10.1186/s12915-016-0341-7 (PMC5282848; doi:10.1186/s12915-016-0341-7)

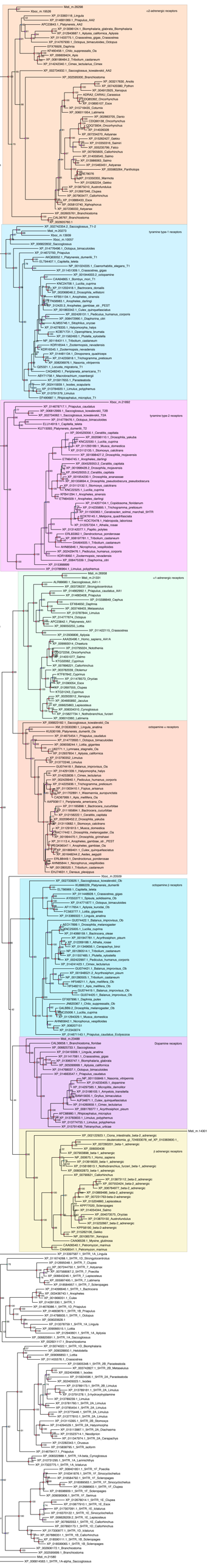

Supplement: Additional file 1: — Maximum likelihood tree of adrenergic, octopamine, and tyramine receptors. Bootstrap support values are shown. This tree contains all investigated GPCRs. The tree was rooted on 5HT receptor sequences. Sub-trees are shown in Additional files 2, 3, 4, 5, 6, 7, and 8. (PDF 118 kb) [file 12915_2016_341_MOESM1_ESM.pdf]

## $\alpha$ 1-adrenergic receptors

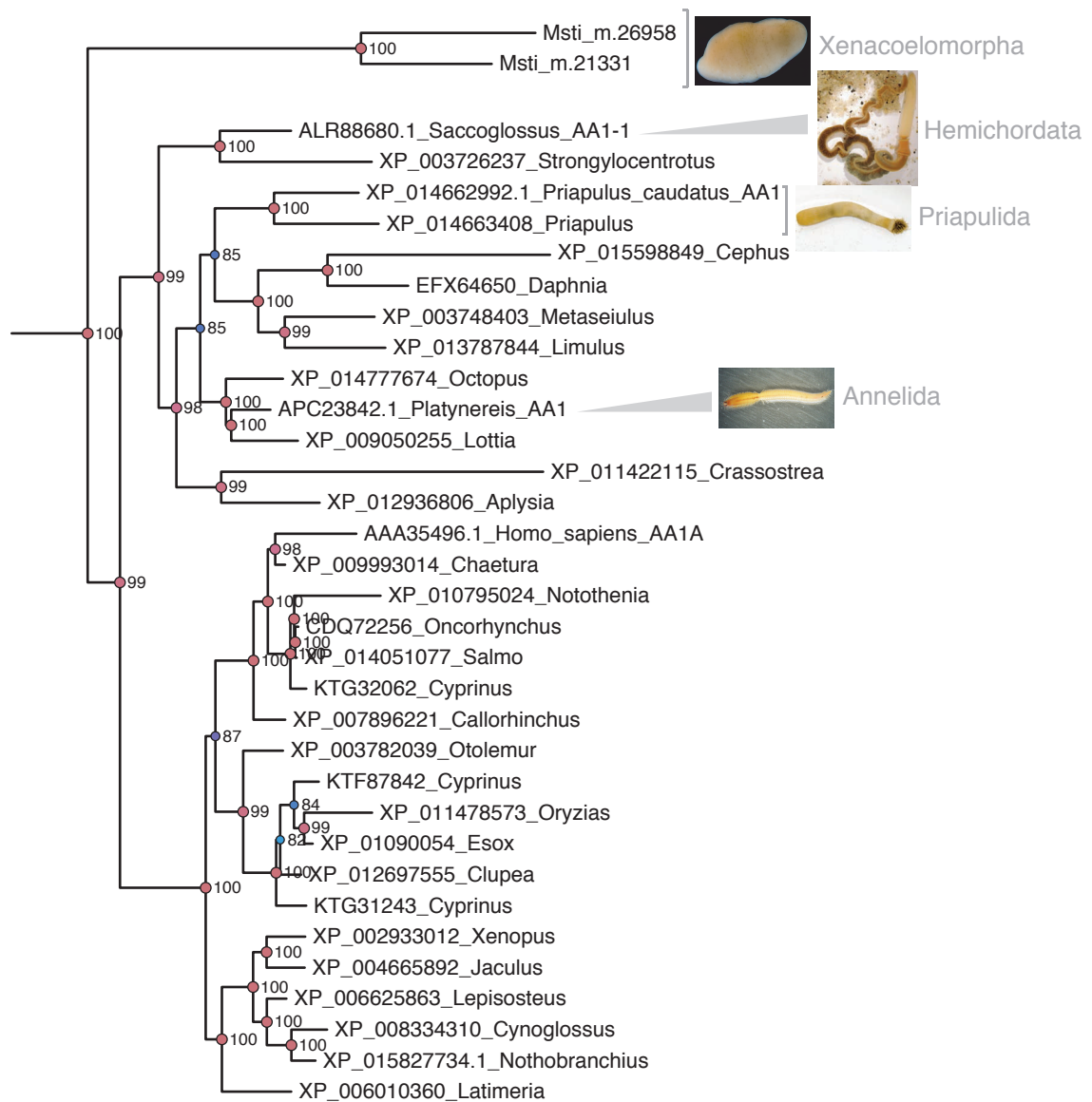

Supplement: Additional file 2: — Maximum likelihood tree of α1-adrenergic receptors. Bootstrap support values are shown for selected nodes. This tree is part of a larger tree containing all investigated GPCRs. (PDF 16992 kb) [file 12915_2016_341_MOESM2_ESM.pdf]

## $\alpha$ 2-adrenergic receptors

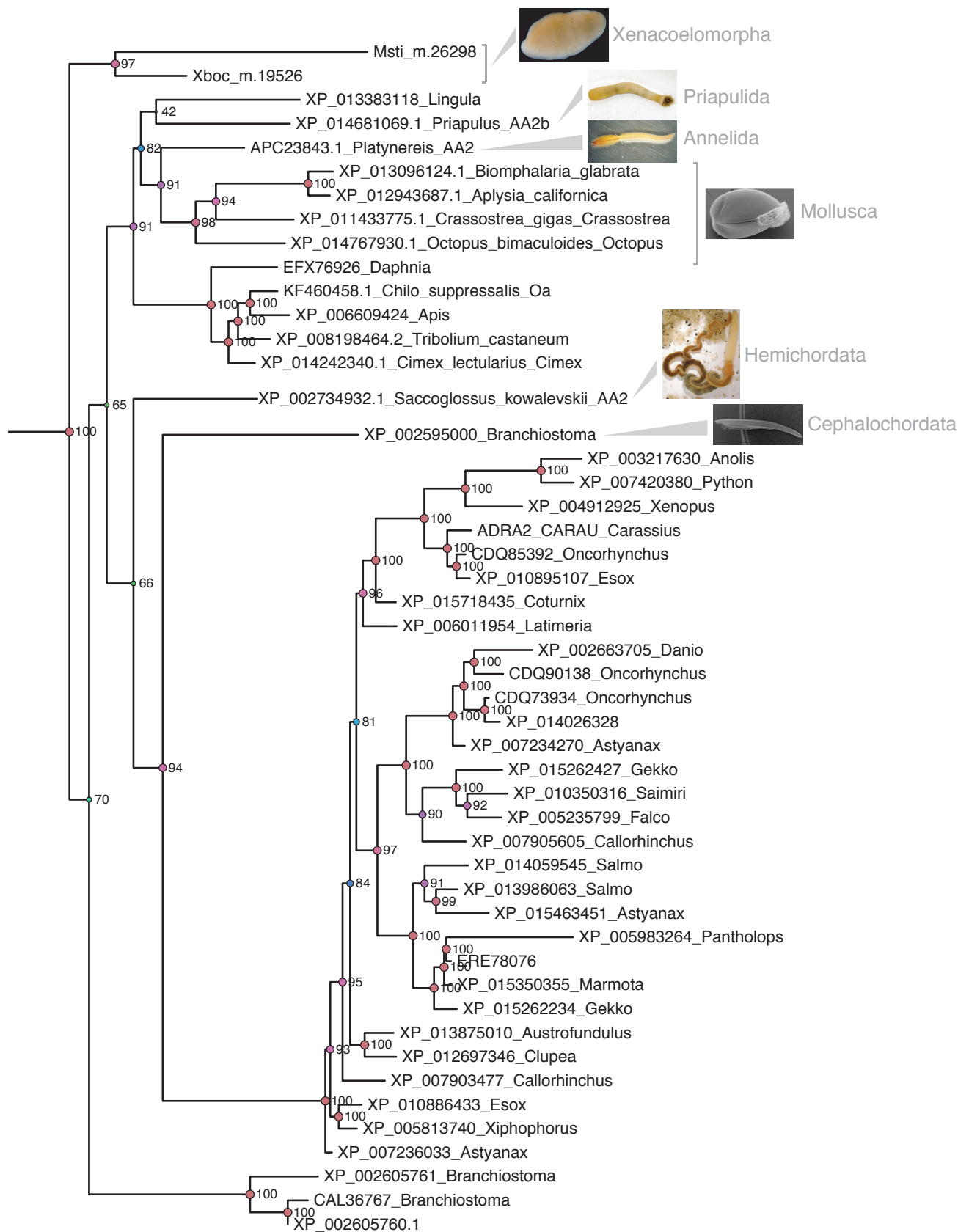

Supplement: Additional file 3: — Maximum likelihood tree of α2-adrenergic receptors. Bootstrap support values are shown for selected nodes. This tree is part of a larger tree containing all investigated GPCRs. (PDF 17168 kb) [file 12915_2016_341_MOESM3_ESM.pdf]

β-adrenergic receptors

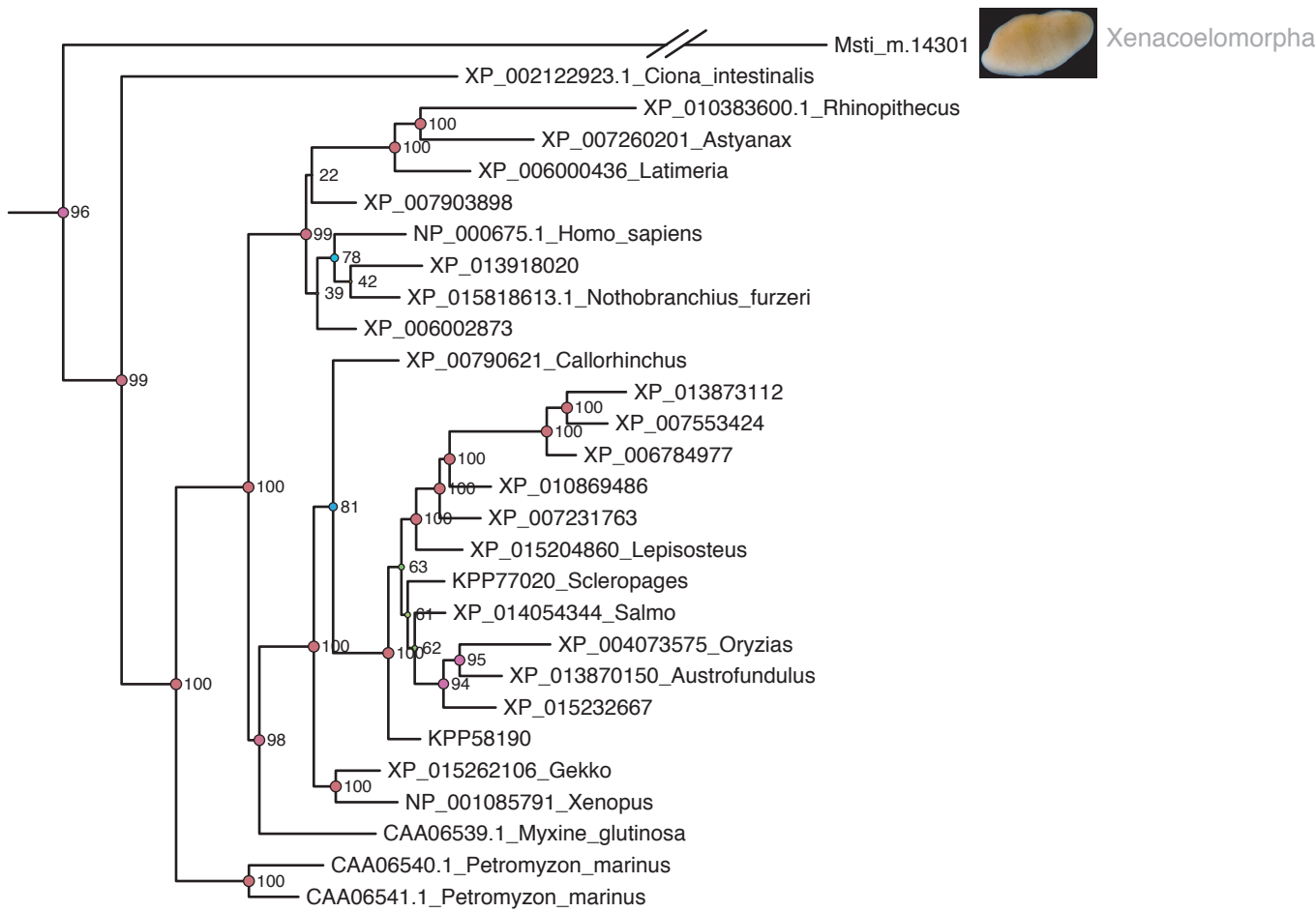

Supplement: Additional file 4: — Maximum likelihood tree of β-adrenergic receptors. Bootstrap support values are shown for some nodes of interest. This tree is part of a larger tree containing all investigated GPCRs. (PDF 759 kb) [file 12915_2016_341_MOESM4_ESM.pdf]

## Tyramine-1 receptors

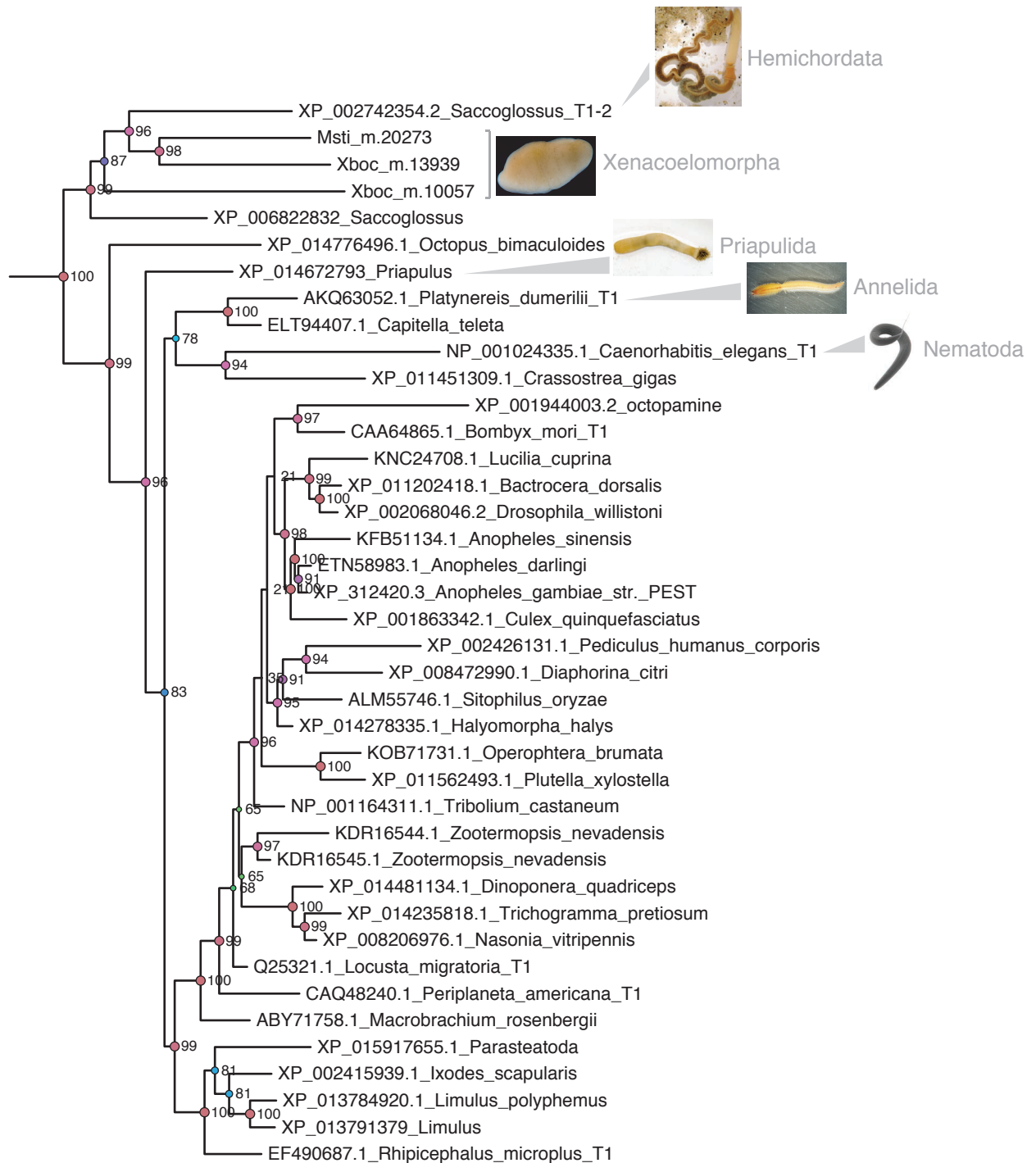

Supplement: Additional file 5: — Maximum likelihood tree of tyramine type 1 receptors. Bootstrap support values are shown for selected nodes. This tree is part of a larger tree containing all investigated GPCRs. The identifiers of deorphanized tyramine receptors were tagged with _T1. (PDF 17028 kb) [file 12915_2016_341_MOESM5_ESM.pdf]

## Tyramine-2 receptors

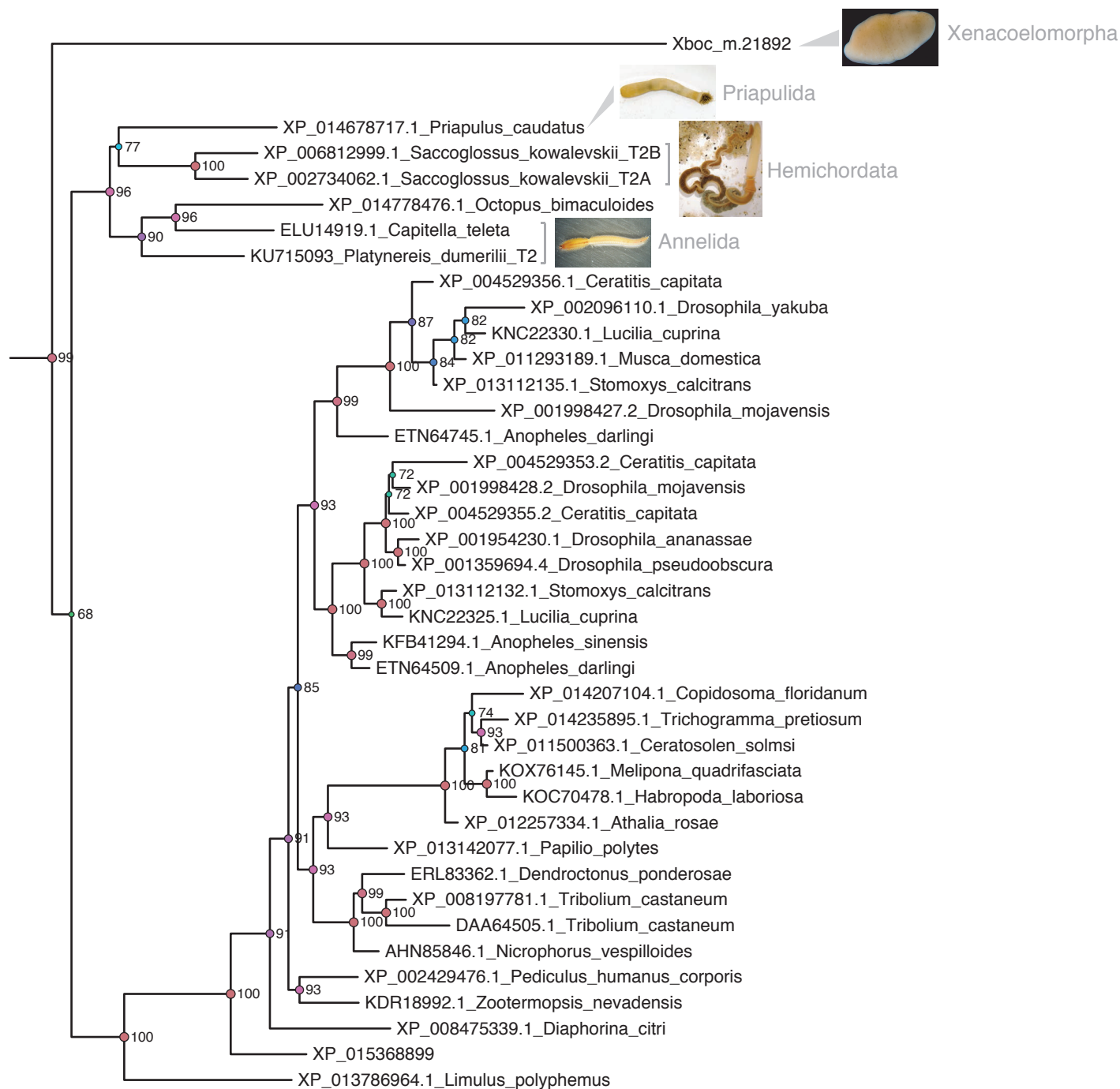

Supplement: Additional file 6: — Maximum likelihood tree of tyramine type 2 receptors. Bootstrap support values are shown for selected nodes. This tree is part of a larger tree containing all investigated GPCRs. The identifiers of deorphanized tyramine receptors were tagged with _T2. (PDF 17007 kb) [file 12915_2016_341_MOESM6_ESM.pdf]

## Octopamin- $\alpha$ receptors

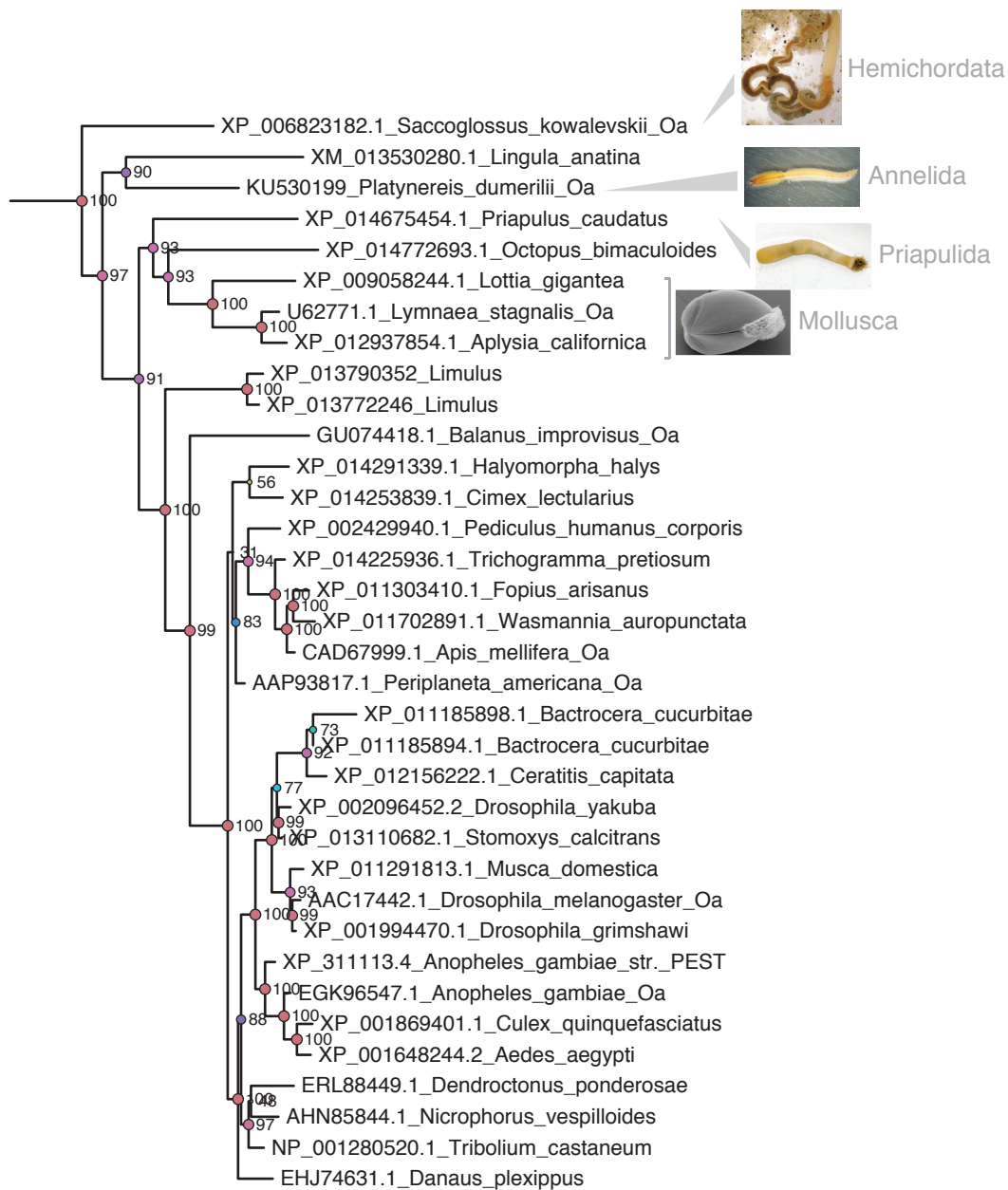

Supplement: Additional file 7: — Maximum likelihood tree of octopamine-α receptors. Bootstrap support values are shown for selected nodes. This tree is part of a larger tree containing all investigated GPCRs. The identifiers of deorphanized octopamine receptors were tagged with _Oa. (PDF 16730 kb) [file 12915_2016_341_MOESM7_ESM.pdf]

## Octopamin- $\beta$ receptors

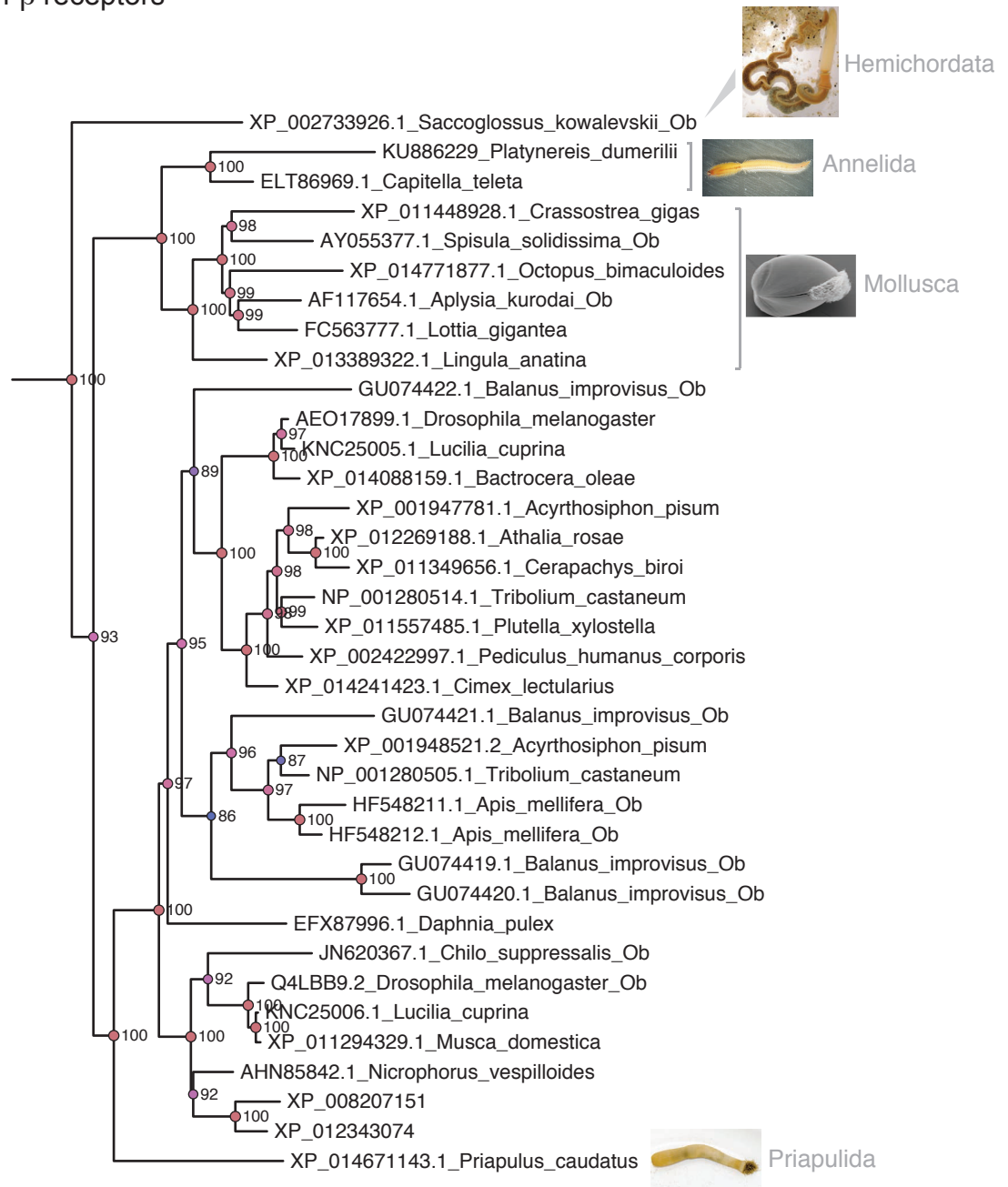

Supplement: Additional file 8: — Maximum likelihood tree of octopamine-β receptors. Bootstrap support values are shown for selected nodes. This tree is part of a larger tree containing all investigated GPCRs. The identifiers of deorphanized octopamine receptors were tagged with _Ob. (PDF 16730 kb) [file 12915_2016_341_MOESM8_ESM.pdf]

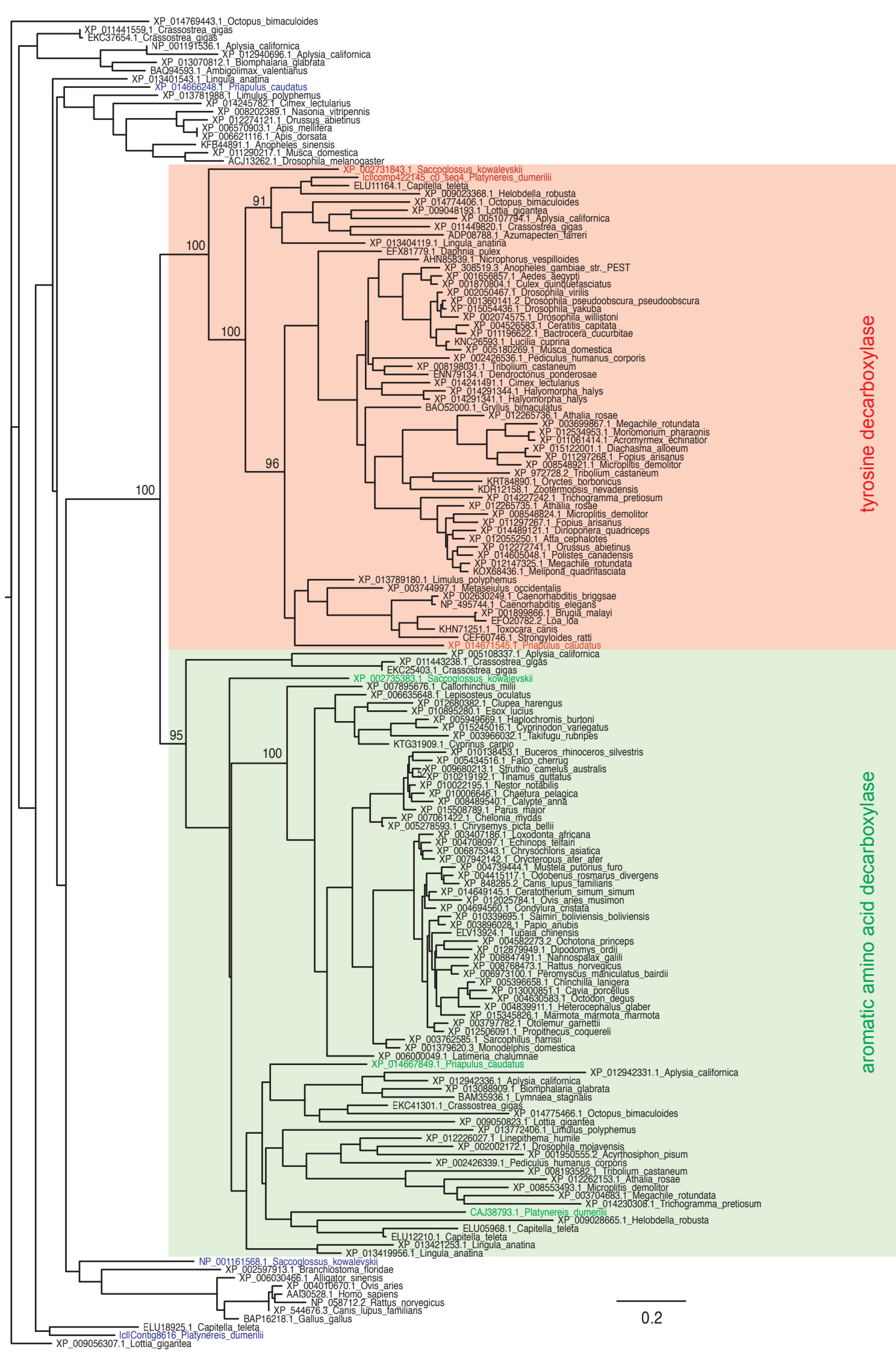

tyrosine decarboxylase

aromatic amino acid decarboxylase

0.2

Supplement: Additional file 9: — Maximum likelihood tree of tyrosine decarboxylase and aromatic amino acid decarboxylase enzymes. Bootstrap support values are shown for selected nodes. P. dumerilii, P. caudatus, and S. kowalevskii sequences are highlighted in color. The Caenorhabditis elegans tyrosine decarboxylase was experimentally shown to be required for tyramine biosynthesis [32]. (PDF 566 kb) [file 12915_2016_341_MOESM9_ESM.pdf]

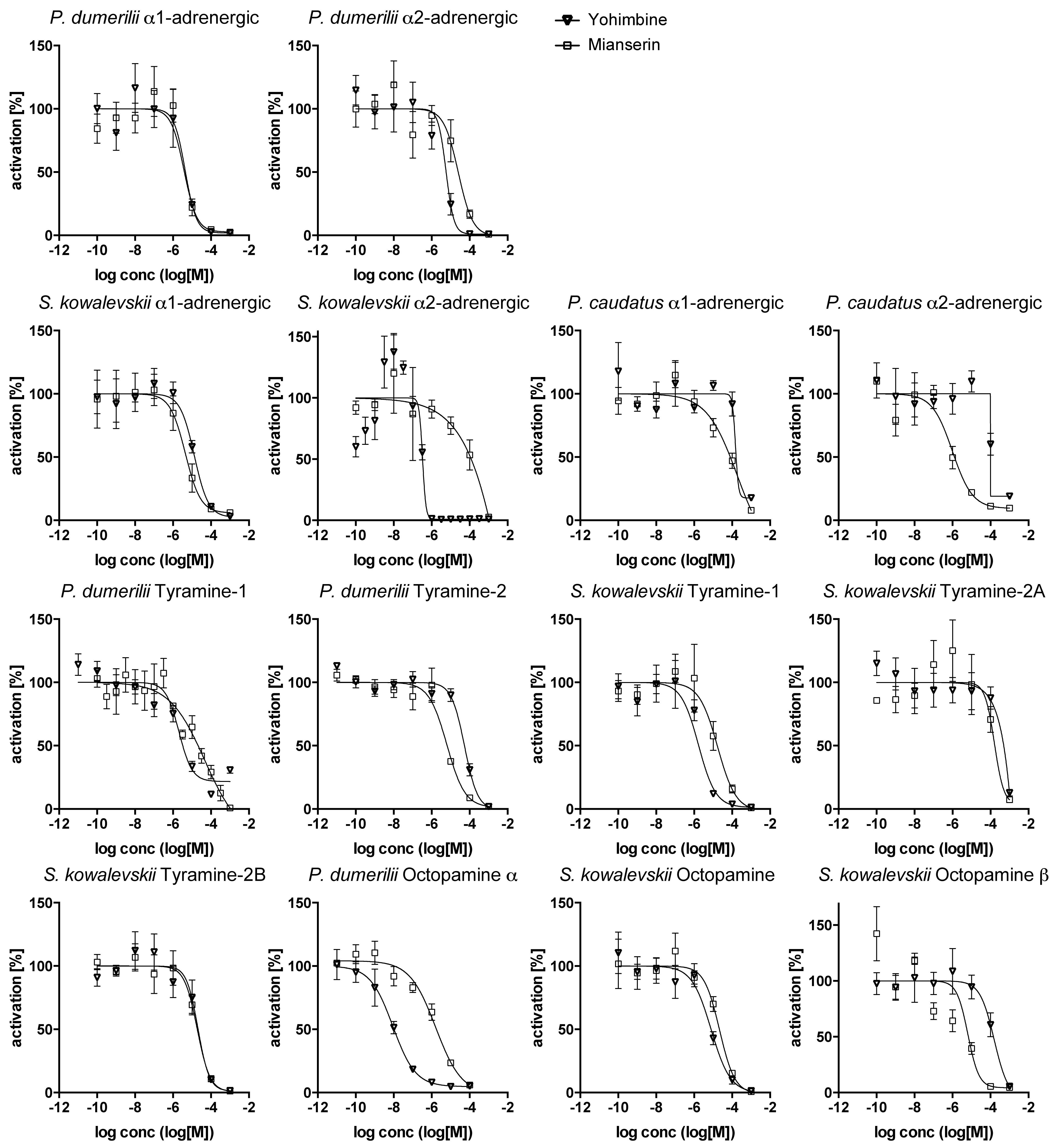

Supplement: Additional file 10: — Dose–response curves of adrenergic, tyramine, and octopamine receptors from P. dumerilii, P. caudatus, and S. kowalevskii treated with varying concentrations of inhibitors. Data, representing luminescence units relative to the maximum of the fitted dose–response curves, are shown as mean ± SEM (n = 3). IC50 values are listed in Table 1. (TIF 956 kb) [file 12915_2016_341_MOESM10_ESM.tif]
